# It's not All Doom and Gloom: Prune Belly Syndrome Associated with VACTERL 

**DOI:** 10.21699/jns.v5i3.337

**Published:** 2016-07-03

**Authors:** Karim Awad, Anupam Lall

**Affiliations:** 1 Department of Paediatric Surgery, Newcastle Upon Tyne Hospitals, Newcastle Upon Tyne, UK; 2 Ain Shams University Hospitals, Egypt

**Keywords:** Prune Belly Syndrome, VACTERL, Tracheo-esophageal fistula

## Abstract

Prune belly syndrome is a rare abnormality; its association with VACTERL is even rarer. This association has been reported in literature a few times since first reported in 1993 and so far the majority have either been stillbirths or died shortly after birth. We present a case of Prune belly syndrome associated with VACTERL who is now one year old.

## INTRODUCTION

Serious congenital malformations cause early fetal loss as they are aborted or miscarried at an early gestational age. Two such examples of developmental defects are Prune belly syndrome (PBS) and VACTERL association. The concurrence of these two syndromes is extremely rare and is generally incompatible with life.[1-5] The incidence of 1.5% was reported for the occurrence of PBS with full VACTERL association and 17.5% for the three components of the VACTERL association [1]. No definite aetiology has been proven, but a defect in mesodermal differentiation, in early first trimester, has been suggested for these two syndromes [2]. We report a neonate of with VACTERL association who also had the features of prune belly syndrome.

## CASE REPORT

A male neonate delivered by a normal vaginal delivery at 38 weeks of gestation presented for antenatally diagnosed hydronephrosis and maternal polyhydramnios. He required a very brief period of intermittent positive pressure ventilation and transferred to Special care baby unit after weaning off the ventilator. At birth, the following components composing of both VACTERL and Prune Belly syndrome were noted (Table1). 

**Figure:**
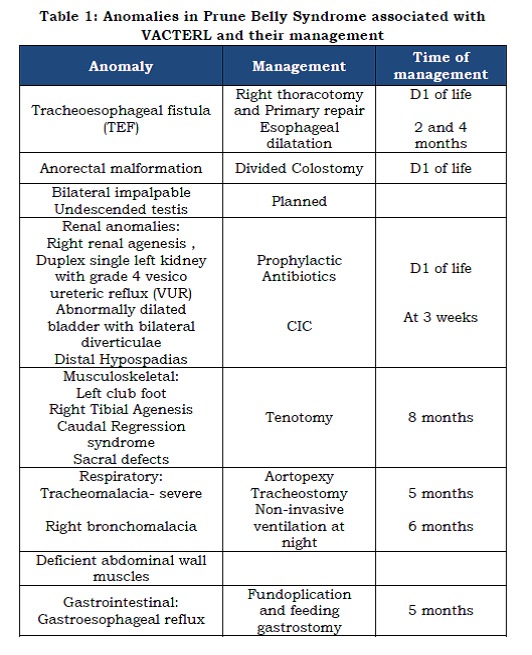
Table 1: Anomalies in Prune Belly Syndrome associated with VACTERL and their management

In summary, all the components of VACTERL association were present except cardiac defect. In addition, bilateral undescended testis; deficient abdominal wall muscles and urological anomalies formed the triad of prune belly syndrome.

He is now one year old, neuro-developmentally normal child, under the care of multidisciplinary team due to the complex nature of his anomalies. He is mainly gastrostomy fed, but takes small amount of pureed diet orally. He remains on 3-4 hourly CIC and prophylactic antibiotics. He is due for corrective hip surgery and bilateral orchidopexy this year.

## DISCUSSION

VACTERL association incidence is 1 in 10,000 to 1 in 40,000 [1], only 1.5% present with the full blown picture while 17.5% present with only 3 components of the association [2]. Prune belly syndrome is less common, with an incidence of 3.8 in 1,000,000 [1]. Hence the chance of having both these anomalies is extremely rare. There has been no solid evidence with regards to the etiology of prune belly syndrome and some of the proposed theories are: Transient bladder outlet obstruction; the second theory is based on mesodermal arrest. Though transient bladder outlet obstruction may be able to explain findings of PBS, yet it fails to explain the VACTERL association. In our case we think the second theory could explain the possible association between PBS and VACTERL association [3].

This concordance of prune belly syndrome with VACTERL association has been reported a few times in literature as shown in Table 2. All the reported cases have been either still births or died shortly after birth. Our baby has survived his surgeries and is now one year old. He is neuro-developmentally normal and being followed up closely by a Multi-disciplinary team involving (Paediatric surgery and Urology, Orthopaedic surgery, Plastic surgery, Respiratory Physicians, Dietetics and Physiotherapists).

**Figure:**
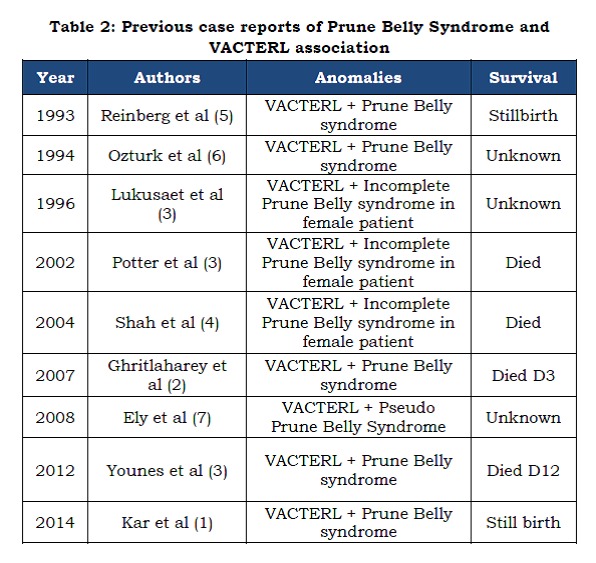
Table 2: Previous case reports of Prune Belly Syndrome and VACTERL association.

## Footnotes

**Source of Support:** Nil

**Conflict of Interest:** Nil
